# The Clinical Course of Alcohol Use Disorder Depicted by Digital Biomarkers

**DOI:** 10.3389/fdgth.2021.732049

**Published:** 2021-12-07

**Authors:** Andreas Zetterström, Markku D. Hämäläinen, Maria Winkvist, Marcus Söderquist, Patrik Öhagen, Karl Andersson, Fred Nyberg

**Affiliations:** ^1^Kontigo Care AB, Uppsala, Sweden; ^2^Uppsala Clinical Research Center, Uppsala Science Park, Uppsala, Sweden; ^3^Rudbeck Laboratory, Department of Immunology, Genetics and Pathology, Uppsala University, Uppsala, Sweden; ^4^Ridgeview Instruments AB, Vänge, Sweden; ^5^Department of Pharmaceutical Biosciences, Uppsala University, Uppsala, Sweden

**Keywords:** digital biomarker, recovery and exacerbation index, addiction monitoring index, maximum time between tests, identification of a relapse

## Abstract

**Aims:** This study introduces new digital biomarkers to be used as precise, objective tools to measure and describe the clinical course of patients with alcohol use disorder (AUD).

**Methods:** An algorithm is outlined for the calculation of a new digital biomarker, the recovery and exacerbation index (REI), which describes the current trend in a patient's clinical course of AUD. A threshold applied to the REI identifies the starting point and the length of an exacerbation event (EE). The disease patterns and periodicity are described by the number, length, and distance between EEs. The algorithms were tested on data from patients from previous clinical trials (*n* = 51) and clinical practice (*n* = 1,717).

**Results:** Our study indicates that the digital biomarker-based description of the clinical course of AUD might be superior to the traditional self-reported relapse/remission concept and conventional biomarkers due to higher data quality (alcohol measured) and time resolution. We found that EEs and the REI introduce distinct tools to identify qualitative and quantitative differences in drinking patterns (drinks per drinking day, phosphatidyl ethanol levels, weekday and holiday patterns) and effect of treatment time.

**Conclusions:** This study indicates that the disease state—level, trend and periodicity—can be mathematically described and visualized with digital biomarkers, thereby improving knowledge about the clinical course of AUD and enabling clinical decision-making and adaptive care. The algorithms provide a basis for machine-learning-driven research that might also be applied for other disorders where daily data are available from digital health systems.

## Introduction

In most fields of medicine, objective measurements provide an indispensable basis for any kind of treatment. For addictive disorders, there is still a lack of precise, objective tools to measure the disease (1). Treatment outcomes are often modest, especially if the desired outcome is long-term, permanent recovery; for alcohol use disorder (AUD), success rates of treatment can be as low as 10% (2), although rates are higher if abstinence is not defined as outcome goal (3).

In the biomedical field it is widely accepted that AUD can be defined as a chronic, relapsing disorder characterized by compulsive drug seeking behavior and continued use, despite well-known harmful consequences, which will lead to long-lasting alterations in the brain (4). In this context it is worth mentioning that some researchers disagree with scientists describing drug addiction as a chronic condition (5, 6). Their concerns are that this formulation privileges biological aspects of dependence to the detriment of psychological and social contributions, and that, accordingly, resources will only be focused on patients in treatment—excluding the vast majority of still functional individuals with addictive disorders (7), who are the main cause of alcohol related costs. Another controverse is related to the objective and goal of treatment: total abstinence and/or reduced/controlled drinking (8). Independently of the above, long-term measurement-based daily monitoring of the clinical course (alcohol use) should be the key for selecting and planning of treatment.

Patients in treatment for AUD can display very large variation in the pattern of lapse/relapse over time (9). It is also well known that binge drinking during weekends is part of social drinking pattern (10, 11). Lapse and relapse can be defined in numerous ways ranging from “any drinking” to 4-10 drinks per drinking day, but no definition has proven better than another (12). Lapse is often defined as a short period of drinking surrounded by longer periods of sobriety, while relapse is seen as a return to old heavy drinking patterns. The dichotomous self-reporting-based sober/relapse view of the disease has been criticized (1, 2) but is still the common way to characterize the outcome of the disease. Based on modern biomarkers (13, 14), continuous monitoring of sobriety with a transdermal sensor (15) and breathalyzer-based eHealth systems (16), self-reported data have been shown to be of poor quality due to underreporting and overreporting (17, 18). Modern biomarkers (e.g., phosphatidyl ethanol, PEth) can accurately measure the level of illness, but they lack the necessary time resolution to measure current trends.

Maisto et al. (1) performed a literature study on the relapse construct and found that it had little research and clinical value. Instead, they argued that measurement-based, near-real-time monitoring of AUD would benefit clinical decision-making and provide knowledge about AUD in general. Modern eHealth systems enable place-unbound continuous monitoring of the clinical course of patients with AUD by measuring the use of alcohol using digital questionnaires and connected transdermal sensors (19–21) or breathalyzers (22, 23). Breathalyzer connected to mobile devices and the cloud has been clinically studied for contingency management (24, 25), monitoring of drunk-driving (26), general monitoring of sobriety of AUD patients (22) and for detailed mapping and machine learning prediction of alcohol use (24). Regarding breathalyzers, measurement values and test compliance patterns have also been compiled into digital biomarkers, which are reported as sensitive tools to identify daily changes in drinking patterns and thus represent the current state or illness level (16, 23, 27).

In this study, we separate the concept of the current trend—recovery or exacerbation—from the current state. Using detailed daily time-series of breathalyzer test results and the pattern of omitted tests it is possible to continuously quantify the sobriety/compliance status of the patient. The current trend can be expressed as a numerical value, and we call it the recovery and exacerbation index (REI). By applying a threshold, we can identify an exacerbation event (EE), which is a negative change in the state of the patient. We use this new terminology rather than the familiar lapse/relapse concept to emphasize the fundamental conceptual difference. We exemplify the use of REI and EEs on eHealth data from two previous clinical studies with AUD patients in Sweden. In the Methods section, we first describe the details on how the recovery and exacerbation index was constructed and how we identified a threshold for the identification of exacerbation events, and then in the Results section, we describe how—together with the addiction monitoring index (AMI)—they were used to describe the clinical course of 51 patients. Finally, we display the average clinical course of 1,717 patients from clinical practice in relation to weekdays, time of year and treatment time.

## Materials and Methods

### Clinical Data and Real Life Evidence

To examine and validate the performance of REI and EEs, we used data from a previously reported clinical trial (16) (NCT03195894). These patients attended the regular care based on motivation conversation and CBT based relapse prevention therapy at Department of Addiction Psychiatry at Uppsala University Hospital in Uppsala combined with therapeutic medication (Disulfiram, Acamprosate, Naltrexone, Nalmefene) or the 12-step aftercare program (one afternoon/week or 1 day/month) at the rehabilitation centre Nämndemansgården. The baseline alcohol use disorder identification test [AUDIT, mean 26.2, SD 6.2 (28)] and short alcohol dependence data [SADD, mean 18.7, SD 7.9 (29)] indicates that these patients had a severe to medium alcohol dependence. We included all patients with eHealth data (*n* = 51: Females/Males: *n* = 19/32, average age 50.0/53.8 years, standard deviation 7.2/8.2 years) and included data up to 365 days resulting in 13,472 patient days. Most of these patients (*n* = 48), including patients with controlled drinking as a goal (*n* = 13), also provided data on drinks per drinking day (DDD, *n* = 4,617). A more limited number of patients (*n* = 39) also provided PEth data.

The patients performed 2–5 daily scheduled breathalyzer tests. The patients received a notification in the mobile phone when it was time to perform a test, and they were given a time window (usually 1–2 h) in which they could perform the test. If they did not perform the test within the scheduled time window, the test was considered as omitted. The results were sent to a cloud-based portal where the caregiver could view the raw results as well as refined data in the form of digital biomarkers, primarily the Addiction Monitoring Index (AMI), see below.

To verify the pattern in time-related periodicities and seasonal patterns in EEs, we also analyzed data collected from clinical practice during 2015–2020 for 1,717 patients stored as anonymized data in Kontigo Care AB's proprietary database. To make the treatment timescale comparable for these two data sets, we used data collected up to day 390, which resulted in a dataset of 241,099 patient days. The mean breathalyzer test performance compliance was 66% of the scheduled tests for both datasets.

### Ethics Approval

The data used were collected from two clinical trials approved 27 September 2015 by the Regional Ethics Committee of Uppsala, Sweden and performed in accordance with the Declaration of Helsinki. The two trials were jointly registered at ClinicalTrials.gov (NCT03195894). Written informed consent was obtained from all participating subjects in the clinical trials. The Regional Ethics Committee of Uppsala, Sweden was asked for approval to use anonymized data (by the company). They answered that such an approval is not needed as it does not constitute personal data due to full anonymization.

### Addiction Monitoring Index (AMI)

As mentioned above, the addiction monitoring index (AMI) is a digital biomarker based on measurement data from an eHealth system for addictive disorders. We previously described its construction, validated it against a chemical biomarker (PEth) and compared it to self-reporting (16, 23). AMI is calculated from results of performed scheduled breathalyzer tests (over/under a 0.2 per mille blood alcohol content limit), and the pattern of omitted scheduled breathalyzer tests. An omitted test is given an imputed value that is lowered for each consecutive omitted test. This means that drinking can be detected from both test results containing alcohol and omitted tests. Another key component of the AMI algorithm is exponential smoothing, i.e., each day's value is constructed from the raw value for the day itself and the smoothed value from previous days (history):


$$\begin{array}{lll} s_{0} & = & x_{0}\\ s_{\text{t}} & = & ax_{\text{t}}+\left(1-a\right)s_{\text{t}-1},t>\;0 \end{array}$$

where a (alpha) is the smoothing factor, i.e., the relative amount of present-day value vs. history.

The digital biomarker AMI in its original implementation had an alpha of 0.21, a level chosen to obtain an optimal balance between history and current state from a treatment perspective. Other values of alpha are possible. A lower alpha will smooth out short-term events and instead provide a long-term perspective. A higher alpha will disregard the long-term perspective and highlight the short-term events. Comparing short-term to long-term perspectives is a common technique applied in other time-series-based domains, e.g., technical analysis of stock markets. AMI lends itself naturally to this kind of analysis—it already incorporates a smoothing algorithm with an adjustable smoothing factor. Moreover, in this study, we used a 0.05‰ blood alcohol content (BAC) threshold [converted from breath alcohol content (BrAC) values by multiplication by 2.0] to define drinking when computing the AMI-21. The 0.05‰ BAC-threshold used to detect drinking is based on the assumption that median statistics is not influenced by secret drinking and therefore we calculated the level of quantification as median + 10 times the interquartile range (*n* = 650,000; median = 0.006; inter quartile range = 0.004; → 0.006 + 10 ^*^ 0.004 = 0.046 < 0.05). This modification leads to a higher sensitivity to detect secret drinking compared with the 0.2‰ used in the original implementation. In this study, we used the following AMI implementations: AMI-6 (alpha = 6, BAC threshold = 0.05‰), AMI-12 (alpha = 12, BAC threshold = 0.05‰), AMI-21 (alpha = 21, BAC threshold = 0.05‰) and AMI-45 (alpha = 45, BAC threshold = 0.05‰).

### Maximum Time Between Tests (MTBT)

The digital biomarker maximum time between tests (MTBT) is the longest time (h) between two tests during a single day and is used to monitor test compliance (27).

### Construction of REI and Identification of EEs

**Figure 1:**
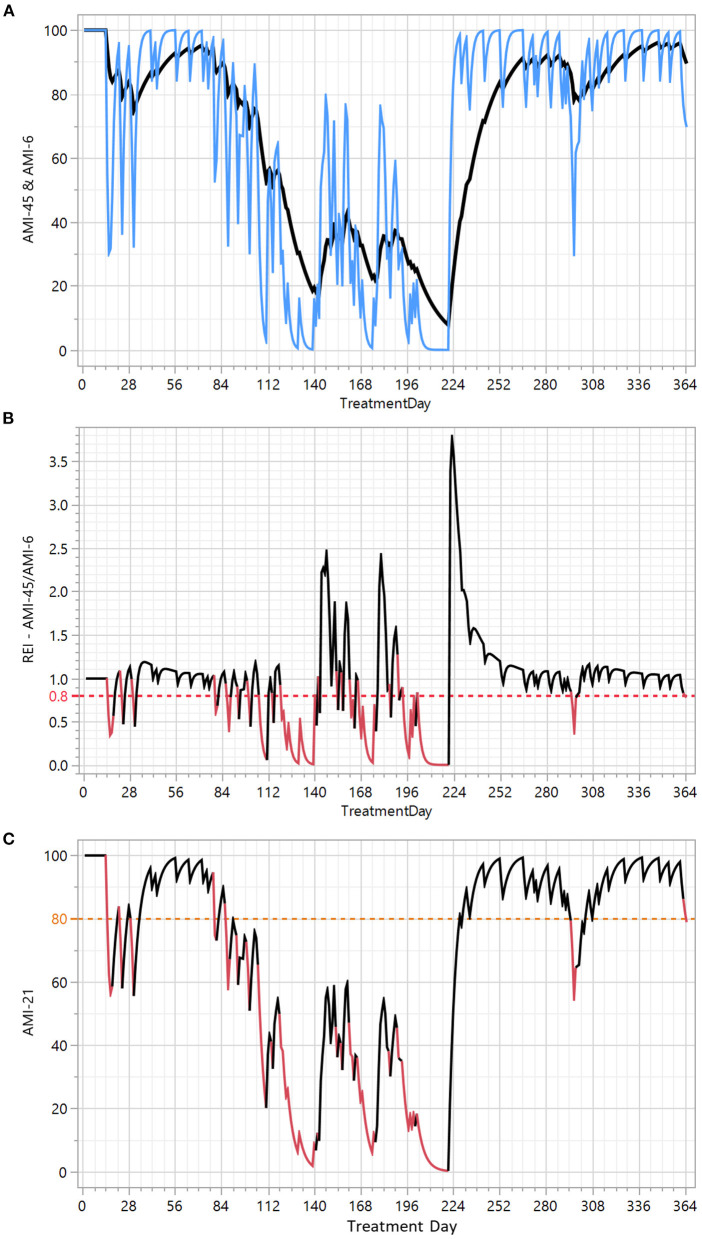
Construction of the recovery and exacerbation index (REI) and exacerbation events (EEs) depicted with patient A. X axis: treatment time in days. Y axes: **(A)** The rapid variation in the clinical course is captured in AMI-45 (blue) and the baseline is defined by AMI-6 (black). **(B)** REI is the ratio (AMI-45/AMI-6 ratio) used to identify exacerbation events. The red line in **(B)** denotes the 0.8 REI threshold used for the detection of EE's displayed as red line segments in the original addiction monitoring index (AMI-21) in **(C)**.

We calculated AMI with different smoothing factors (alpha): AMI-45 (very short-term), AMI-21, AMI-12, and AMI-6 (very long-term) and constructed different REIs as ratios between a shorter-term and longer-term AMI. To understand the characteristics of different types of REIs, we calculated 7 AMI ratios (45/6, 45/12, 45/21, 21/12, 21/6, 12/6, 12/6) and studied how ratios and thresholds handled our goal to detect changes in breath alcohol content measurements and compliance for a set of patients. The 45/6 ratio was selected as it gave the best contrast between baseline and an exacerbation event and thus best served our goal to identify the current trend independent of the current level. To depict the construction of the REI, we display AMI data (6, 21, 45) and the AMI-45/6 ratio (REI) for patient A (Figure 1). AMI-45 rapidly reacts to changes in sobriety/compliance. AMI-06 reacts slowly and acts as baseline. AMI-21 shows a medium change and is currently presented to the therapist in the system (with the difference that in the system, the therapist can choose the BAC threshold).

**Figure 2:**
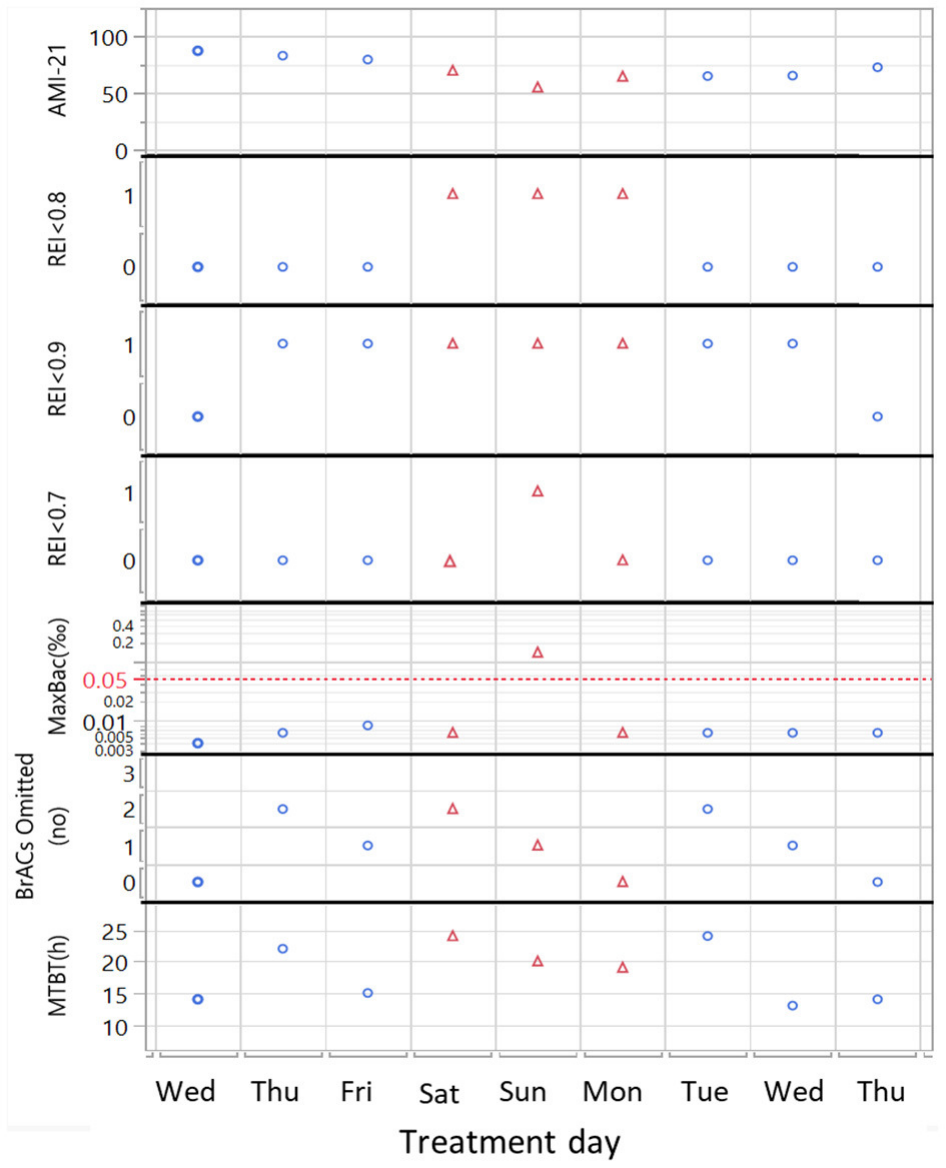
Schematic illustration of REI threshold selection exemplified with a weekend relapse for a patient with 3 scheduled tests/day. The algorithm based on the optimal REI < 0.8 threshold identifies 3 Exacerbation Events (EE = 1, red triangles). REI < 0.9 threshold is too sensitive and identifies 7 EE's, while the REI < 0.7 threshold identifies only 1. Only the Sunday EE is triggered by identified alcohol use (MaxBAC > 0.05‰), while the 2 other days during the same relapse are triggered by poor test compliance. X axis: treatment time in days. Y axis from top: the addiction monitoring index (AMI-21), REI in < 0.8, <0.9 and <0.7 bins, where 1 denotes that an EE event has occurred, highest blood alcohol content measurement that day [MaxBAC (‰)], no of omitted tests (out of 3 scheduled) and maximum time between tests [MTBT (h)].

When AMI-45 is above AMI-6, the patient is faring better from a short-term perspective (AMI-45) than from a long-term perspective (AMI-6). The AMI-45/AMI-6 ratio provides a numerical measure of this comparison between the short-term and long-term perspectives—independent of the current level. When we succeeded in constructing the digital biomarker REI that describes the current trend, we continued by searching for an optimal threshold of the REI to define EEs. We used a graphical (Figure 2) and a statistical method. A schematic illustration of the graphical method using a weekend relapse (Figure 2), shows that the selected REI < 0.8 threshold identifies 3 days in EE, while REI < 0.9 identifies 7 (too sensitive) and REI < 0.7 only 1 day. This graphical method (Figure 2) was based on an overlay display of alcohol measurements (MaxBAC) and test compliance (no of omitted tests, MTBT) and applied to a well-known set of the 51 patients from a previous clinical trial. We qualitatively assessed that a threshold of 0.7 seemed to identify too few and too short EEs and that a threshold of 0.9 seemed to identify too many and too long EEs, with 0.8 as a sensible compromise.

A second, more quantitative way to select an optimal threshold was to use drinks per drinking day (DDD, *n* = 4,641) as a response in ANOVA. For each REI threshold (0.05–1.1), the 4,641 days with DDD data were divided into 2 bins: belonging to EE [1] or not [0]. We then performed ANOVA using the 0/1 variable (with and without using patient as a fixed factor) and studied the mean difference in DDD using the F-ratios for all 17 models. The F-ratio is low at both low and high REI thresholds and is high in the range REI 0.6–0.8. A cut-off of 0.8 captured more weekend relapses (e.g., see Figure 2) that were otherwise lost if a lower threshold was used. Since the qualitative graphical analysis and ANOVA both supported REI < 0.8, this threshold was chosen.

With the REI <0.8 threshold, we can define the start and end points of an exacerbation event (EE), and each EE can be characterized by its length (EE_Length_), which is the number of days below the threshold. The distance to the end of the previous EE in days (EE_Dist_) is an additional interesting characteristic. The number of EEs (EE_no_), the percentage of days in EE (%EE), and the average length of EEs (meanEE_Length_) and distance (meanEE_Dist_) give a compiled overview of the drinking pattern of the patient (Table 1).

**Table 1:** Background characteristics and exacerbation event data for 7 patients.

**Patient code**	**Treatment days (no)**	**PEth (umol/L) mean**	**DDD (no) mean**	**AMI-21 (mean)**	**MTBT(h) mean**	**%EE**	**EE count (no)**	**EE Length (days) mean**	**EE Length (days) max**	**Time since previous EE (days) mean**
B	365	<0.05	0.03	98	14	4%	10	1.4	4	37.9
C	359	<0.05	0.81	90	19	12%	27	1.6	10	10.1
D	353	0.61	2.21	68	16	18%	32	2.0	9	9.0
A	365	1.07	0.57	68	43	26%	21	4.6	22	12.8
E	363	0.34	3.82	39	19	39%	60	2.4	13	3.7
F	340	0.30	4.24	42	169	54%	19	9.7	41	7.9
G	314	1.23	8.65	16	201	63%	25	7.9	50	5.0

*Data on the following are included: biomarker phosphatidyl ethanol (PEth), self-reported drinks per drinking day (DDD), addiction monitoring index (AMI-21) and maximum time between tests (MTBT)*.

### Statistical Analysis

The statistical analysis if weekdays, week numbers and treatment day bins significantly influence EE was performed using logistic regression with repeated measures by patient ID (*n* = 13,472 [clinical trial], *n* = 241,099 [company database]). The visualization of the differences is based on means and bars showing the 95% confidence interval of %EE (see corresponding figures).

## Results

### Overview of the Clinical Course for 51 Patients

**Figure 3:**
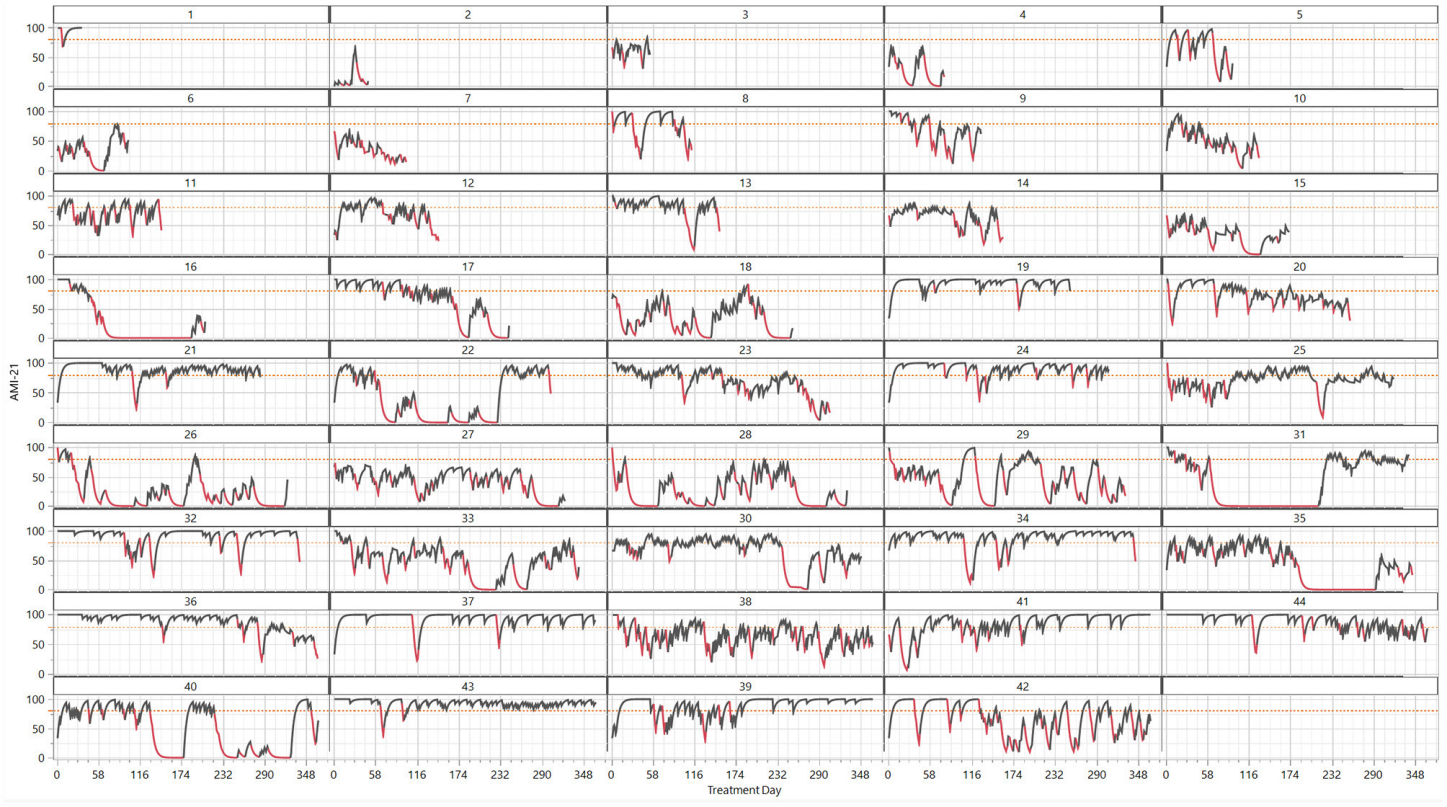
Clinical course of 44 patients shown as treatment day time series (x-axis) of the digital biomarkers addiction monitoring index (AMI-21, y-axis) and exacerbation events of a length ≥ 2 (EE = 1, red; EE = 0, black). The patients are arranged by increasing treatment days, starting with early dropouts.

**Figure 4:**
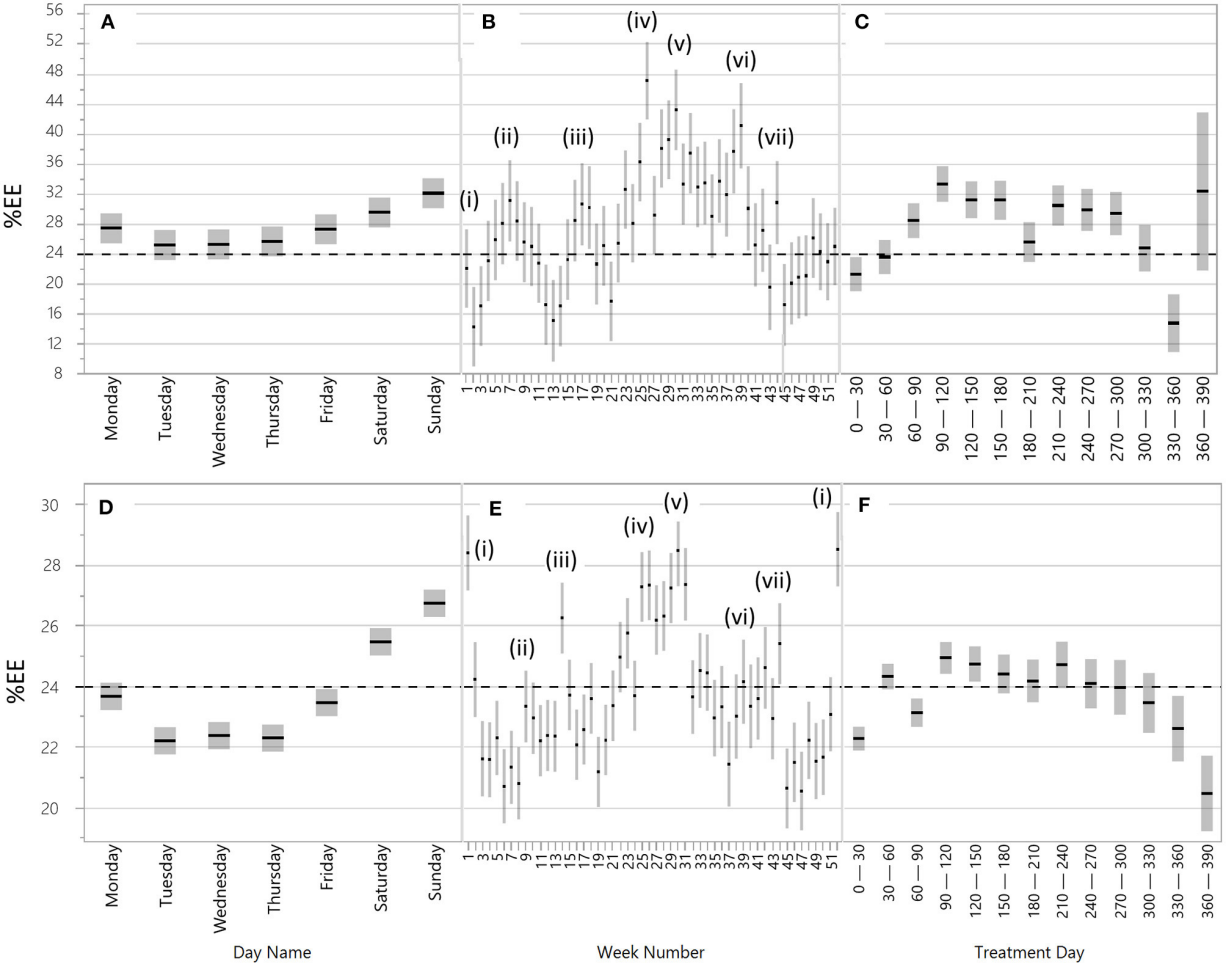
Time-related periodicities and seasonal patterns in exacerbation events (%EE) for the 51 patients from the clinical trials **(A–C)** and for 1,717 patients from clinical practice **(D–F)**. Periodicities and seasonal patterns are related to **(A)** and **(D)** weekdays (day name); **(B)** and **(E)** week numbers, i.e., holidays; and **(C)** and **(F)** time from treatment start (treatment days). Explanations of (i–vii) in **(B)** and **(E)**: (i) New year and Christmas, (ii) Swedish school winter vacation weeks, (iii) Easter, (iv) Swedish Midsummer, (v) summer vacations, (vi) Swedish moose hunt, and (vii) Halloween and Swedish school autumn vacation week. The line at y = 24 is included to enhance the comparison between the groups of patients **(A–C)** vs. **(D–F)**. Note that the y-axis has a different range in **(A–C)** vs. **(D–F)**.

**Figure 5:**
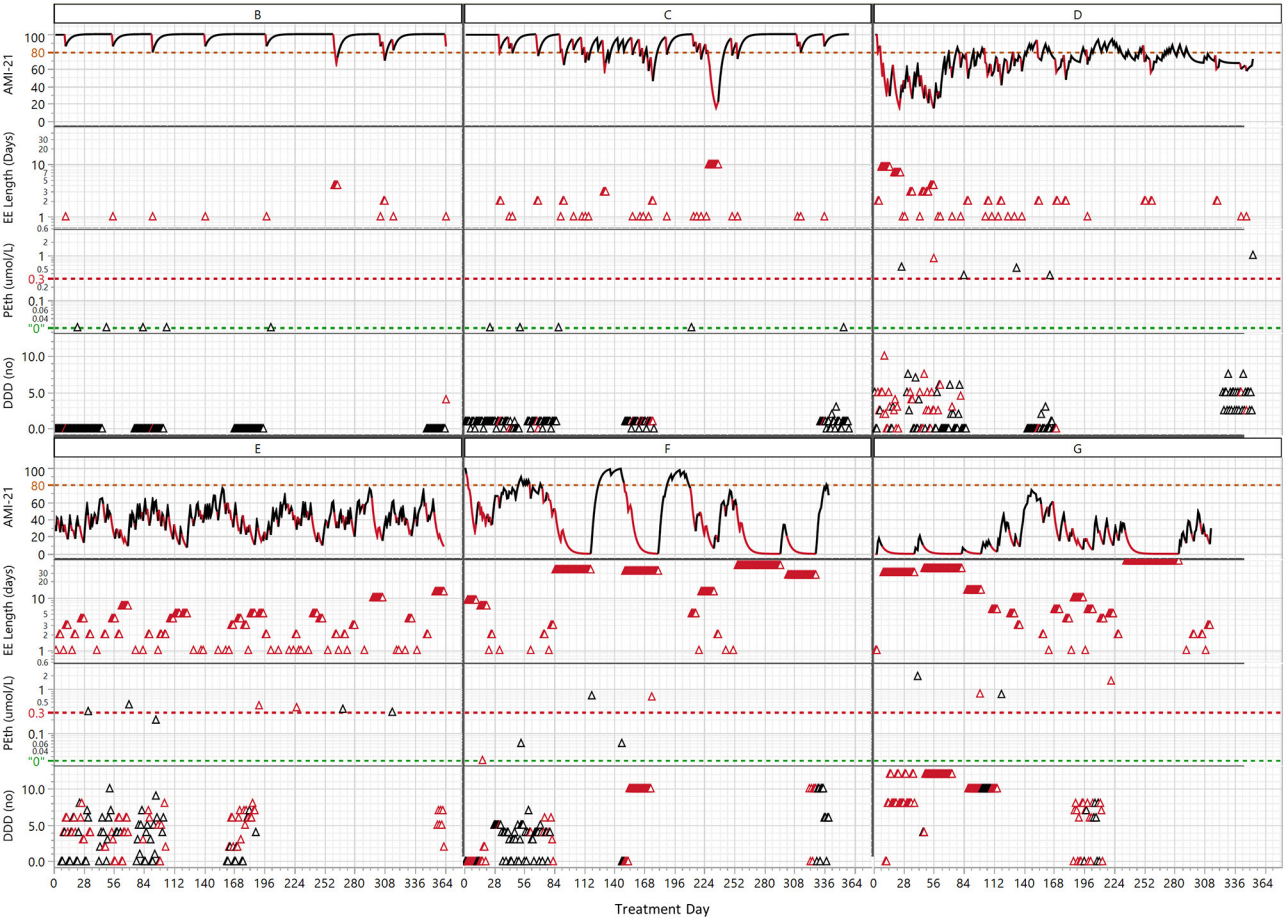
The clinical course of the 6 patients listed in Table 1 interpreted with both the digital biomarker addiction monitoring index (AMI-21) and exacerbation events (EE length) combined with the biomarker phosphatidyl ethanol (PEth) and drinks per drinking day (DDD) arranged in order of increasing average EE percentages (see Table 1). The red color indicates days classified with the REI as EEs. The green line at “0” denotes the analytical detection limit of PEth, and the red line at 0.3 μmol/L is considered indicative of heavy drinking. In the EE length graph, the same total length of the EE is plotted for each day on the x-axis, i.e., a 10-day-long EE has 10 red triangles on 10 consecutive treatment days.

The digital biomarker-based description of the clinical course of the patients showed extreme variation over time in EE frequency and length (Figures 1, 3, 5) and was influenced both by short- and long-term periodicities (Figure 4). Part of the variation in drinking episodes as captured in EE could be explained by known increased risk of “social” addictive behavior and increases with treatment time from 0 to 3–4 months: there were significant short-term periodicities that correlated with weekdays (Figure 4A: *p* < 10^−4^, *n* = 13,472; Figure 4D: *p* < 10^−4^, *n* = 241,199; higher prevalence of EEs on Sat and Sun), seasonal patterns (Figure 4B: *p* < 10^−4^, *n* = 13,472; Figure 4E: *p* < 10^−4^, *n* = 241,099) %EE used to visualize the variation pattern peaks at Christmas and the New Year [i], Swedish school winter vacation week [ii], Easter [iii], Swedish Midsummer [iv], summer vacation [v], Swedish moose hunt [vi], Swedish school autumn vacation week [vii]) and treatment time (Figure 4C: *p* < 10^−4^, *n* = 13,472; Figure 4F: *p* < 10^−4^, *n* = 241,099).

### In-depth Analysis of the Clinical Course for Seven Patients

To explore how digital biomarkers can describe the clinical course of AUD patients in a detailed way, we analyzed seven patients (A–G) in depth. These patients were chosen because they had a long treatment period and represented distinctive drinking patterns. A compiled tabular view of all 7 patients is presented in Table 1. Patient A was graphically presented in Figure 1; patients B-G are graphically presented in Figure 5.

Table 1 summarizes the background and EE data for patients A-G in order of increasing percentage of EE_days_ (4–63%). The average drinks per drinking day (DDD) varied from virtually zero (0.03) to >8, and for two patients, none of the PEth measurements indicated drinking. The abstinent (B) and extremely controlled drinkers (C) had higher average AMI levels than the rest of the patients. The number of EEs was similar (20–30) for B, C, F and G, but the average EE_length_ distinguished B (~2) as “light” and F and G (8–10) as “heavy” drinkers. F and G were also characterized by short average times between EE occasions.

The AMI and EE of the 6 patients B-F (Figure 5) revealed drinking patterns that were not visible in self-reported (DDD) or PEth data. First, there was large variation in the intensity of drinking over time, and depending on the exact point in the time series when the PEth/DDD measurement was taken, the patient might be judged as abstinent or a heavy drinker. Digital biomarkers, however, can discern drinking patterns in the clinical course with high time resolution. For example the extremely controlled drinking of patient C (1 DDD) was not detected with PEth but was clearly seen with digital biomarkes. The periodicity varied between the patients. There was a pattern of frequent low/medium-intensity drinking (C, D, E) compared to prevalently month-long drinking periods for F and G. This type of extreme difference in periodicity was seen when comparing E and F, which had similar average DDD and PEth results. E interestingly had two overlapping periodicities of different frequencies (week, ~2 months), while F has month(s)-long drinking periods followed by month-long sober periods. Patients F and G had more than half of the days classified as being in EEs, which was reflected in the average (~8–10 days) and maximum (41, 50 days) EE lengths.

## Discussion

Traditional definitions of relapse usually rely on self-reporting in combination with thresholds. This means that relapse is seen as a disease “state” or “level”. If relapse is seen as a level of illness regardless of the previous level, the somewhat difficult question arises: for how long must the patient have been “sober”—and at what level of sobriety—in order to qualify for having a relapse if drinking starts again? (1). We believe that these ideas are the consequence of the low-quality input data rather than constituting the optimal way to view the clinical course. The possibility of measuring the level of illness with high time resolution makes a separation of level and trend logical. Accordingly, a “relapse”—a term we replace by “exacerbation event”—can happen at any level of disease. We emphasize that the trend alone does not tell us how badly ill the patient is; it is independent of illness level. The trend tells us if the patient, from a short-term perspective, is faring better or worse than from a long-term perspective. It has been suggested that recovery is usually a continuous process rather than a discrete event, and the occurrence of lapses and relapses should be considered a natural part of the disease (7, 30). Accordingly, AUD should be managed as a chronic condition with proactive care to maintain behavioral change. Ecological momentary analysis and interventions that allow rapid action have been suggested as a remedy in the treatment of addictive disorders (1, 31). The new insights into the clinical course provided by digital biomarkers strengthen evidence for this view of AUD as a “difficult to treat” condition on a continuous scale and provide useful tools for applying this knowledge in clinical practice.

The clear connection between %EE-based clinical course of AUD and short- and long-term periodicities in typical social drinking patterns provides an indirect verification of the quality of the digital biomarkers. Our results clearly indicate that addiction is more active at times when drinking is more socially accepted (10, 11) and when treatment is unavailable. The correlation between %EE and increasing treatment time until 4 months shows that EE also captures the known difficulty of sustaining a behavioral change in the first months of therapy (30). It is important to understand that average %EE captures the time from the start of an exacerbation event until an improvement starts. Although %EE values of 20–30% are unexpectedly high, recent eHealth-based monitoring of the sobriety of outpatients supports our findings (15, 16). The minor differences in weekly periodicities between the 51 patients from the clinical trials and the validation set of 1,717 patients from the company database are related to differences in the time frame (0–2 vs. 0–6 years) and geography (Uppsala area vs. whole of Sweden), which influenced the position of non-fixed holidays (Easter, Winter school holiday, Moose hunt). The higher %EE level of the 51 patients from the clinical study could be explained by the fact that this cohort included patients with more severe addiction (hospital care vs. municipal care). Approximately 25% of the 51-patient cohort had controlled drinking as a goal, something that may also have influenced the higher level and variability in %EE over the year.

It is obvious from the current study that the REI algorithm can identify exacerbation events (EEs) from a wide variety of AMI profiles. The algorithm adjusts the identification of EEs to the average current sobriety/compliance level of the patient. This means that the sensitivity for detecting deviations is higher for patients with no current positive test results and very good compliance. For these patients, a 24-h long MTBT can be detected as a deviation—a possible increased risk of false positive warnings. For patients with higher variability in test compliance (and positive breathalyzer tests), longer periods without tests (i.e., ~48 h) are needed before an EE is detected. Furthermore, EEs are more difficult to identify when the patient systematically avoids positive tests by omission. When the AMI is calculated, a positive breathalyzer test immediately receives a higher negative weight than missed tests (16), which makes the detection of secret drinking slower when patients omit tests; after 48 h of constant omission of tests, however, there is equal negative weight regardless of positive or omitted tests. This makes the initial AMI decline slower when a drinking episode starts by omitting tests, as opposed to when it starts with a positive test. A slower AMI decline makes the EE appear at a slightly later time or not be classified as an EE. The abovementioned situation with high variability and systematic omission of tests leading to fewer EEs was reflected and detected by decreased AMI levels and higher MTBT. As shown in Figure 1, REI is close to zero for patients with long EEs, and when followed by rapid improvement in recovery, REI rapidly climbs far above 1. This makes it more difficult to identify EEs after such a deep dive/rapid climb combination, and in some cases, the algorithm can even consider a positive test result to be “bad business as usual” compared to periods with several week-long relapses, i.e., it mainly happens when the AMI level is very low (e.g., <60; Figure 2 [3]). This concept of verified drinking not being considered an EE might seem radical, but we are convinced that when the AMI level is so low, the patient drinks more or less every day; in that situation, a positive breathalyzer test only means that the patient performed the test, whereas most of the previous tests were omitted.

In some situations, especially if the patient exhibits a very short-frequency periodic pattern, there could be a risk of potentially identifying too many EEs—and if implemented in the system, this would lead to unwanted therapeutic actions detrimental to the therapist/patient alliance. However, if the actions taken by the therapist are of a less resource-demanding type (SMS/mail or phone calls), the cost can be minimized. Increased attention would seldom be considered by the patient as negative, and a call might interrupt a process that could potentially have serious and costly consequences. When building machine learning models for forecasting an EE_length_ ≥ 2 days is considered as a “relapse” (Figure 3).

The automatic extraction of REI characteristics for a patient includes, in addition to the occurrence of EEs, the frequency, number, length and time between lapses/relapses. This means that we can categorize patients into different behavioral groups (weekend drinking, periodic drinking, etc.) based on high-quality measurement-based data that enable more detailed modeling of the disease pattern. This pattern can then be followed over time, and when patients move from one group into another, the therapy form can be adjusted to best fit the current disease state.

The method, in the presented form, monitors alcohol use and test compliance to form a continuous view of the clinical course. It is well known that a key factor to relapse prevention is to understand both its gradual onset (32, 33) and the three characteristic signs of relapse (emotional, mental and physical). Relapse often begins with emotional and mental relapse weeks before a physical relapse when the individual picks up a drink or drug. Understanding the characteristic signs of relapse has been shown to decrease drinking (34) frequency. This means that just monitoring substance use should be combined with monitoring of factors related to emotions and mood, cravings, therapy compliance and daily routines (e.g., eat/sleep/social activities) to be able to early detect an increased risk and prevent relapses and binge drinking.

The National Institute of Drug Abuse (35) in the USA provides a broad view of current literature. They underline that treatment providers need to choose an optimal plan for treatment in consultation with the individual patient. Lapses back to alcohol use suggest that it is necessary to reinstate or adjust treatment, or that alternative treatment should be considered. State of the art eHealth systems where the presented digital biomarkers are used to monitor the clinical course should be equipped with an open infrastructure for communication and for monitoring of cravings/mood/motivation/daily routines and for applying different psychological treatment methods. This would allow the treatment method to be rapidly adjusted to the current patient need.

### Generalization to Other Addictive and Psychiatric Disorders

Digital biomarkers hold great promise for transforming the treatment of not just AUD but all addictive disorders, as well as many psychiatric disorders (36–38). The concept of AMI, REI, and EE can be generalized to any addictive or psychiatric disorder where some kind of behavioral incidents poses a problem, a reduction in the number, gravity and frequency of these incidents signifies recovery from the disease, and where the ultimate goal is to cease having such incidents. These incidents can be measured and managed by an eHealth system by physical measurements as described in our study or by Alessi et al. (40), digital questionnaires (1, 31, 39, 41), actigraphy (38) and other sensors (37), and monitoring of smartphone use (36), and these measurements could then be compiled into AMI, REI, and EE.

## Limitations

In addition to detailed technical limitations discussed above, the method as presented is in this publication is tailored to serve the recovering AUD patient. The data set under analysis contains two categories of patients in care and after-care; one targeting abstinence and one targeting controlled drinking. The applicability of the method to other groups of AUD patients is unknown. It should be understood that the nature of AUD brings exceptional variability in the cohort, meaning that even though this analysis is made on data covering more than 13,000 days from ~50 individuals, it cannot be excluded that not all common causes for relapse are represented in a balanced manner.

Also, whereas the concepts of AMI, REI, and EE should be readily transferable to many other addictive disorders, it has to be recognized that the algorithmic composition needs tailoring in each case.

## Conclusions

The digital biomarker AMI, emanating from a breathalyzer-based eHealth system, enables an algorithmic analysis of the recovery status of alcohol use disorder patients on a continuous scale, in contrast to the traditional binary sober/relapse perspective. This study indicates that the disease state—level, trend and periodicity—can be visualized and mathematically described, thereby improving knowledge about the clinical course of AUD. The REI and EE can provide useful tools for therapists, who could perform adequate therapeutic actions when the REI indicates that the patient is currently in a bad trend or in an EE—a significant improvement for AUD clinical decision-making and the basis for an adaptive treatment design. Further research could aim to construct and empirically test treatment programs adapted to different drinking patterns. The fact that trends and events are algorithmically identified enables the creation of large-scale input data for mathematical/statistical modeling and machine learning, with the goal of predicting adverse events in advance. The AMI and REI algorithms can be applied to any disorder for which daily measurables are available and whose successful treatment requires persistent behavioral change. We conclude that our study indicates great potential for digitalization to bridge the gap between research and clinical practice.

## Data Availability Statement

The data analyzed in this study is subject to the following licenses/restrictions: Permission to display time series profiles of individuals was granted data by Regionala Etikprövningsnämnden in Uppsala, Sweden. Reasonable request to access these datasets should be directed to markku.hamalainen@kontigocare.com.

## Ethics Statement

The studies involving human participants were reviewed and approved by Regionala Etikprövningsnämnden Uppsala, Sweden. The patients/participants provided their written informed consent to participate in this study.

## Author Contributions

AZ invented REI and MH identified optimal threshold to define EE and both of them drafted the article. All authors contributed to the intellectual content and writing to the article.

## Funding

This work was supported by Vinnova, the governmental Swedish Innovation Agency (Grant no 2014-03659).

## Conflict of Interest

MH, AZ, MW, and MS are all employees of Kontigo Care AB. FN is member of the scientific advisory committee of Kontigo Care AB. AZ, MH, and KA are inventors of a patent related to this manuscript. The remaining author declares that the research was conducted in the absence of any commercial or financial relationships that could be construed as a potential conflict of interest.

## Publisher's Note

All claims expressed in this article are solely those of the authors and do not necessarily represent those of their affiliated organizations, or those of the publisher, the editors and the reviewers. Any product that may be evaluated in this article, or claim that may be made by its manufacturer, is not guaranteed or endorsed by the publisher.

## Abbreviations

AUD: Alcohol use disorder
AMI: Addiction monitoring index
REI: Recovery and exacerbation index
EE: Exacerbation event
MTBT: Maximum time between tests (h)
%EE: Proportion of exacerbation days in relation to treatment days.
